# Temperature Variability during Delirium in ICU Patients: An Observational Study

**DOI:** 10.1371/journal.pone.0078923

**Published:** 2013-10-23

**Authors:** Arendina W. van der Kooi, Teus H. Kappen, Rosa J. Raijmakers, Irene J. Zaal, Arjen J. C. Slooter

**Affiliations:** 1 Department of Intensive Care Medicine, University Medical Centre Utrecht, Utrecht, The Netherlands; 2 Department of Anesthesiology, University Medical Centre Utrecht, Utrecht, The Netherlands; Oregon Health and Science University, United States of America

## Abstract

**Introduction:**

Delirium is an acute disturbance of consciousness and cognition. It is a common disorder in the intensive care unit (ICU) and associated with impaired long-term outcome. Despite its frequency and impact, delirium is poorly recognized by ICU-physicians and –nurses using delirium screening tools. A completely new approach to detect delirium is to use monitoring of physiological alterations. Temperature variability, a measure for temperature regulation, could be an interesting component to monitor delirium, but whether temperature regulation is different during ICU delirium has not yet been investigated. The aim of this study was to investigate whether ICU delirium is related to temperature variability. Furthermore, we investigated whether ICU delirium is related to absolute body temperature.

**Methods:**

We included patients who experienced both delirium and delirium free days during ICU stay, based on the Confusion Assessment method for the ICU conducted by a research- physician or –nurse, in combination with inspection of medical records. We excluded patients with conditions affecting thermal regulation or therapies affecting body temperature. Daily temperature variability was determined by computing the mean absolute second derivative of the temperature signal. Temperature variability (primary outcome) and absolute body temperature (secondary outcome) were compared between delirium- and non-delirium days with a linear mixed model and adjusted for daily mean Richmond Agitation and Sedation Scale scores and daily maximum Sequential Organ Failure Assessment scores.

**Results:**

Temperature variability was increased during delirium-days compared to days without delirium (β_unadjusted_=0.007, 95% confidence interval (CI)=0.004 to 0.011, p<0.001). Adjustment for confounders did not alter this result (β_adjusted_=0.005, 95% CI=0.002 to 0.008, p<0.001). Delirium was not associated with absolute body temperature (β_unadjusted_=-0.03, 95% CI=-0.17 to 0.10, p=0.61). This did not change after adjusting for confounders (β_adjusted_=-0.03, 95% CI=-0.17 to 0.10, p=0.63).

**Conclusions:**

Our study suggests that temperature variability is increased during ICU delirium.

## Introduction

Delirium is an acute disturbance of attention, consciousness and cognition that usually fluctuates over time[1]. It is a common disorder in the intensive care unit (ICU), with three different subtypes based on psychomotor behavior: hypoactive, hyperactive and mixed-type delirium[2]. Delirium is associated with higher mortality, longer hospital stay, long-term cognitive impairment and increased costs[3-6]. 

Despite its frequency and impact, recognition of delirium by ICU-physicians is poor (overall sensitivity 29%)[2]. In order to improve early diagnosis and treatment, the Society of Critical Care Medicine and the American Psychiatric Association recommend daily monitoring of delirium in ICU patients[7,8]. Various delirium assessment tools have been developed. Of these, the Confusion Assessment Method for the ICU (CAM-ICU) has the highest sensitivity (80% in meta-analysis)[2,9,10]. In contrast to results from a research setting, the sensitivity of the CAM-ICU showed to be low in routine, daily practice (overall 47%)[11]. This hinders early detection of delirium, and the subsequent delay in treatment may worsen patient outcome[12]. 

A completely new approach to detect delirium is to use monitoring of physiological alterations[13]. Delirium is a manifestation of encephalopathy which may also affect thermoregulation. In delirium tremens, Wernicke encephalopathy, as well as schizophrenia, temperature regulation is disturbed[14-16]. However, temperature variability has never been investigated in ICU patients with and without delirium. The primary aim of this study was to investigate whether ICU delirium is related to temperature variability. We hypothesized that delirium is associated with increased temperature variability. Secondary, we investigated whether ICU delirium is related to absolute body temperature.

## Materials and Methods

### Study Design

In this single-center retrospective cohort study, patients were selected from three prospective studies conducted at the ICU of the University Medical Centre Utrecht (UMCU) between March 2009 and May 2012. These three prospective studies included: the control group of the randomized clinical trial on Rivastigmine [17], The ICU Environment Study [18] and the Epidemiology of ICU Delirium Study (unpublished data, but study results were presented at the European Delirium Association 7^th^ annual meeting, 2012, Bielefeld, Germany). All three studies were approved by the UMCU medical ethics committee. For the trial on Rivastigmine patients gave written informed consent (UMCU medical ethics committee number 08-077), whereas for the latter two studies, the UMCU medical ethics committee waived the need to obtain informed consent (UMCU medical ethics committee number 11-450 and 12-421 respectively). From these three studies patient characteristics and prospective delirium and RASS assessments were obtained. In addition, temperature data was extracted retrospectively from the patient data management system. The local medical ethics committee approved the retrospective study Delirium and Temperature Variability (UMCU medical ethics committee number 11-567). 

### Patients

All medical records of patients included in the three prospective studies mentioned above were re-evaluated for possible inclusion in the present study. All adult patients with at least one delirious and one delirious-free episode during an ICU admission of at least 24 hours were included, except for the following five exclusion criteria: (1) disturbed regulation of body temperature: renal replacement therapy, extra corporal membrane oxygenation, therapeutic hypothermia or admission because of an intoxication; (2) no temperature data in the medical record; (3) persistent delirium or comatose state during the whole ICU admission, which makes comparison of delirium- with non-delirium days impossible; (4) admission because of a neurological- or neurosurgical disease, as it may be difficult to diagnose delirium in these patients; or (5) impossibility to be tested with the CAM-ICU, for example because of an inability to understand Dutch or English. A comatose state was defined as a Glasgow Coma Score lower than 9 or a Richmond Agitation and Sedation Scale (RASS) score lower than minus 3.[19,20] Furthermore, we excluded patients with sepsis throughout their whole ICU admission as well as days with sepsis in other patients. Sepsis was defined as two or more systemic inflammatory response syndrome criteria together with a suspected or proven infection described in the medical record[21]. All included patients were treated with paracetamol 1000 mg 4 times daily, both on days with delirium, as on days without. None of the patients were receiving dexemedatomidine during their ICU admission. 

### Data Collection

In all three original studies, patient data was collected per day and baseline parameters, sepsis parameters, as well as RASS and Sequential Organ Failure Assessment (SOFA) scores were extracted from the medical records[22]. 

Because of suboptimal sensitivity of the CAM-ICU in daily practice [11], delirium was assessed in all three original studies, prospectively, seven days a week, by a research-nurse or –physician. This delirium assessment included the scoring of the CAM-ICU by the research-nurse or –physician prospectively, review of medical records and review of nursing charts including CAM-ICU scores performed twice-daily by bedside nurses. Based on this assessment, the research-nurse or –physician made a daily classification of the mental status of patients as either: (1) awake and non-delirious, (2) delirious or (3) comatose, as defined above. In doubtful cases, a neurologist-intensivist (AJCS) was consulted, who made the final classification. Temperature in the ICU was automatically controlled in every room to be constantly 18 degrees Celsius using a thermostat.

Temperature data was measured every minute in the inguinal crease or rectum with a temperature probe (Respectively YSI 403 or YSI 409B, YSI temperature, Dayon, Ohio, U.S.A.). Measured temperature data was filtered and sampled by the Spacelabs Medical Ultraview® Command Module (Spacelabs healthcare, LLC, Issaquah, WA, U.S.A.). Temperature data was stored at 1 sample per minute in the patient data monitoring system (Metavision, version 5.45.62, iMDsoft, Needham, Massachusetts, U.S.A.). Data analysis and artifact detection was conducted in Matlab (Matlab, version 7.9.0.529, The MathWorks Inc, Natick, Massachusetts U.S.A.). For artifact detection, temperature measurements below 35 degrees were excluded, together with data from the preceding and following 20 minutes, in order to overcome decreases in temperature due to a removed thermometer. Per measurement day at least 144 temperature measurements (10%) had to be available after artifact removal, otherwise that day was excluded for temperature variability analysis.

### Outcome

The primary outcome was temperature variability. Temperature variability was defined as the daily mean absolute second derivative (i.e. acceleration) of the body temperature signal. The secondary outcome was absolute body temperature and this was defined as the daily mean of the body temperature signal. 

### Statistical Analysis

Temperature variability data as well as absolute body temperature data were averaged per day. Data of a particular day was excluded from analysis, when that day a patient was comatose, had only less than 10% of the 24 hour temperature data available, suffered from sepsis or died.

All variables were tested for normality using the Kolmogorov-Smirnov test. Normally distributed variables were presented using mean and standard deviation (Std) , non-normally distributed variables with median and interquartile range (IQR). 

Patients with and without delirium were compared for differences in temperature variability and absolute body temperature, and additionally adjusted for level of activity (mean RASS) and disease severity (maximal SOFA score). Linear mixed models were used to account for clustering of multiple, daily measurement averages per patient. Delirium scores, as well as possible confounders, mean RASS and maximal SOFA scores, were included as fixed effects. All models included a random intercept. Random slopes for the fixed effects were included when the Akaike Information Criterion of that particular model was five points lower than the Akaike Information Criterion of the same model with only a random intercept. The used covariance type for models with only a random intercept was ‘identity’; in all other cases it was ‘unstructured’. Statistical analyses were performed with Statistical Package for the Social Sciences (IBM SPSS Statistics, version 20, Armonk, New York, U.S.A.). A two-tailed p-value less than 0.05 was considered to be statistically significant.

## Results

**Figure 1:**
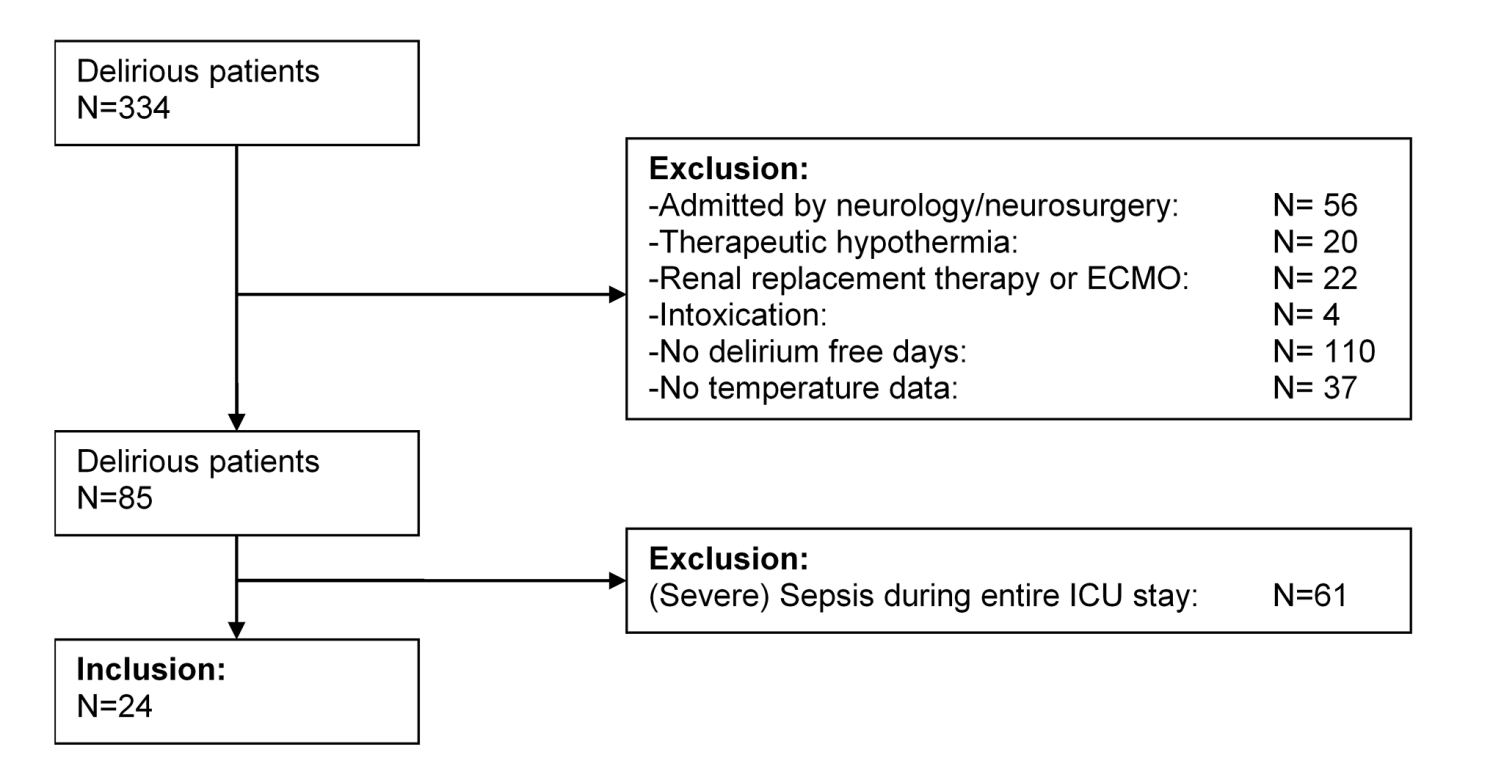
Screening and enrolment. Abbreviation in Figure 1: ECMO=Extra Corporal Membrane Oxygenation.

In total, 334 delirious patients were evaluated of whom 24 patients were included (Figure 1). Characteristics of the included patients are described in Table 1. Nine patients were female. The mean age was 68 years old (Std 14) and mean Acute Physiology and Chronic Health Evaluation IV score was 52 (Std 21). Median length of ICU stay in these patients was 5 days (IQR 3.3 to 9.8). The median number of delirium days in the study population was 2 (IQR 1.0 to 2.0) and the median number of non-delirium days 1 (IQR 1.0 to 2.8). 

**Table 1:** Patient characteristics.

**Case**	**Age**	**Gender**	**Admitting Discipline**	**Apache IV**	**Delirium type**	**Temperature (mean±SD)**		**Temperature variability (mean±SD) 10^-2^ °C/min^2^**		**Number of analysed days (n**)	
						**D**	**ND**	**D**	**ND**	**D**	**ND**
1	37	M	Gen. Surg.	49	Mixed	37.4±0.2	37.6±0.3	1.2±0.4	0.8±0.3	2	7
2	75	M	Int. Med	94	Mixed	36.9±0	37.3±0	1.8± 0.1	0.4± 0	2	1
3	53	F	Card. Surg.	26	Mixed	37.9±0	37.8±0	1.6±0	0.8±0	1	1
4	69	M	Card. Surg.	48	Hypo	36.3±0	36.0±0	1.6±0	1.3±0	1	1
5	68	M	Int. Med	85	Mixed	36.3±0.4	36.1±0.4	1.5± 1.1	1.0±0.4	4	4
6	73	M	Gen. Surg.	41	Mixed	37.0±0.3	36.9±0.2	2.2±0.6	2.0±0.6	9	6
7	78	M	Gen. Surg.	100	Mixed	36.6±0.4	36.8±0.3	1.5±0.5	1.7±0.6	4	6
8	82	M	Gen. Surg.	72	Hypo	36.4±0	37.0±0.1	1.6±0	0.9±0.5	1	2
9	71	M	Gen. Surg.	61	Mixed	37.3±0.3	37.9±0	2.5±1.5	0.3±0	2	1
10	65	M	Gen. Surg.	36	Hypo	36.7±0	37.4±0	3±0	2.6±0	1	1
11	80	M	Card. Surg.	60	Mixed	37.1±0	36.3±0	2±0	1.9±0	1	1
12	75	M	Card. Surg.	76	Mixed	37.7±0.6	37.4±0	2.9±0.6	0.8±0	2	1
13	79	F	Card. Surg.	63	Mixed	36.7±0.1	36.8±0	2.5±0.4	1.5±0.4	2	1
14	53	M	Card. Surg.	81	Mixed	37.2±0.2	37.5±0	2.6±0.2	1.4±0	2	1
15	55	F	Int. Med	38	Mixed	37.1±0.1	37.3±0.1	2.4±0.6	2.7±0.1	2	2
16	74	F	Gen. Surg.	62	Hypo	37.4±0	36.3±0	1.7±0	1.3±0	1	1
17	58	F	Card. Surg.	30	Mixed	36.0±0.2	36.4±0.2	2.2±0.6	2.1±0.1	3	3
18	84	M	Card. Surg.	69	Mixed	36.9±0	36.8±0	3.1±0	1.8±0	1	1
19	75	F	Card. Surg.	62	Mixed	36.6±0	36.2±0	3.6±0	3.4±0	1	1
20	72	F	Card. Surg.	38	Hypo	36.5±0.1	37.0±0	3.9±1.3	1.0±0	3	1
21	35	F	Card. Surg.	58	Hypo	35.9±0	35.8±0	1.0±0	1.3±0	1	1
22	67	M	Gen. Surg.	47	Mixed	36.9±0	37.0±0	1.3±0.5	0.8±0	2	1
23	79	M	Gen. Surg.	88	Mixed	37.0±0.2	36.9±0.4	2.0±1.0	1.9±0	2	3
24	44	F	Gen. Surg.	38	Mixed	36.7±0	37.2±0	3.6±0	1.6±0	1	1

Abbreviations in table 1: Apache IV=Acute Physiology and Chronic Health Evaluation IV score; Card. Surg.=Cardiology and cardiac surgery; D=Delirium; Gen. Surg.=General surgery; Hyper=Hyper active delirium; Hypo=Hypo active delirium; Int. Med.=Internal medicine; Mixed=Mixed type delirium; ND=Non-Delirium. Type of delirium was based on Richmond agitation and sedation scale (RASS) during delirium days: Hyper =always RASS > 0, Hypo= always RASS score < 1, Mixed=not always RASS > 0 or RASS <1.

**Figure 2:**
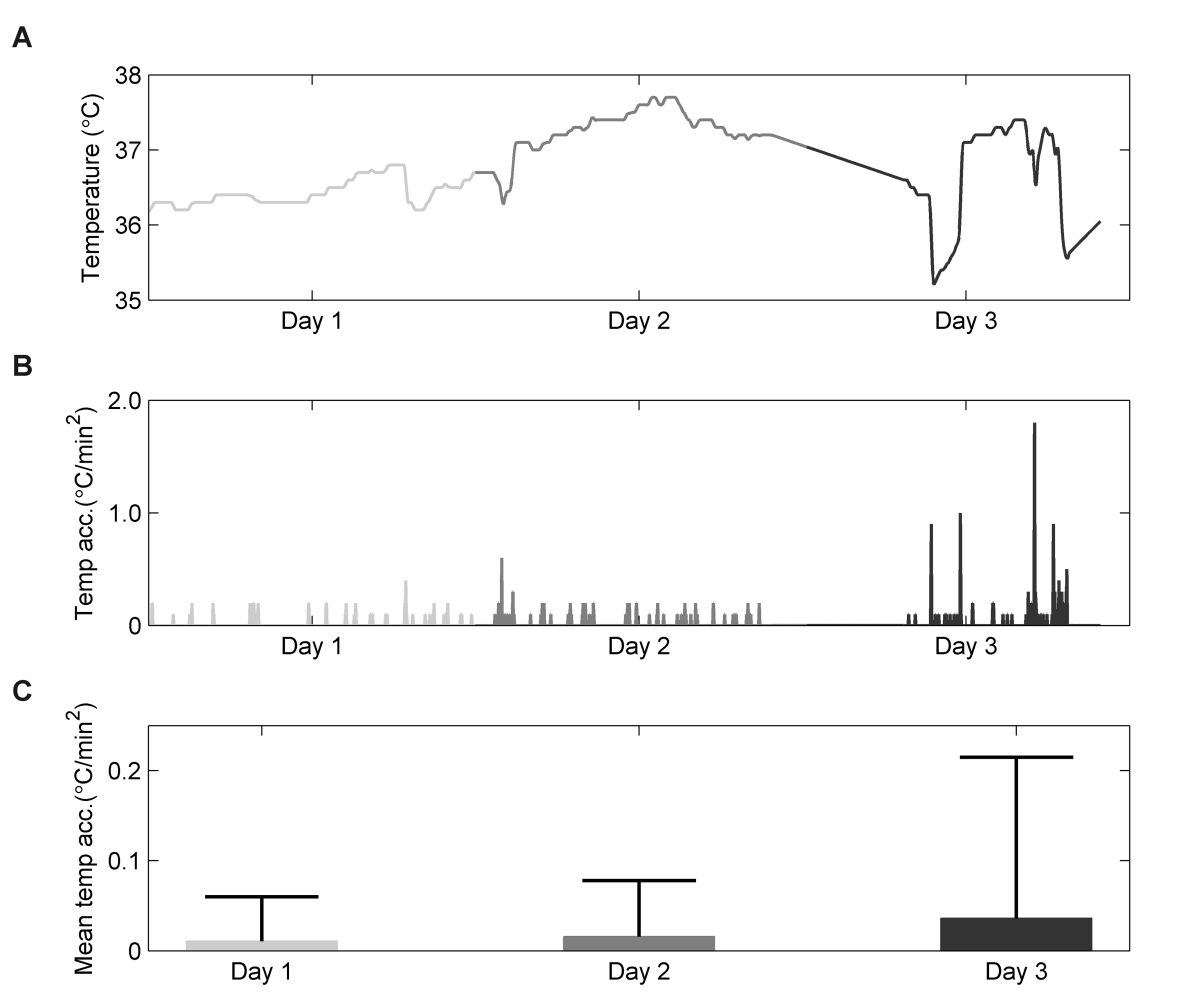
Example of temperature variability determination. Panel A shows the temperature curve, which was corrected for artifacts using linear interpolation. Panel B shows the normalized temperature acceleration and panel C the mean normalized temperature acceleration per day with corresponding standard deviations. Periods of coma are depicted in light grey, periods of no delirium in dark grey, and periods of delirium in black. The whiskers represent the standard deviation.

**Figure 3:**
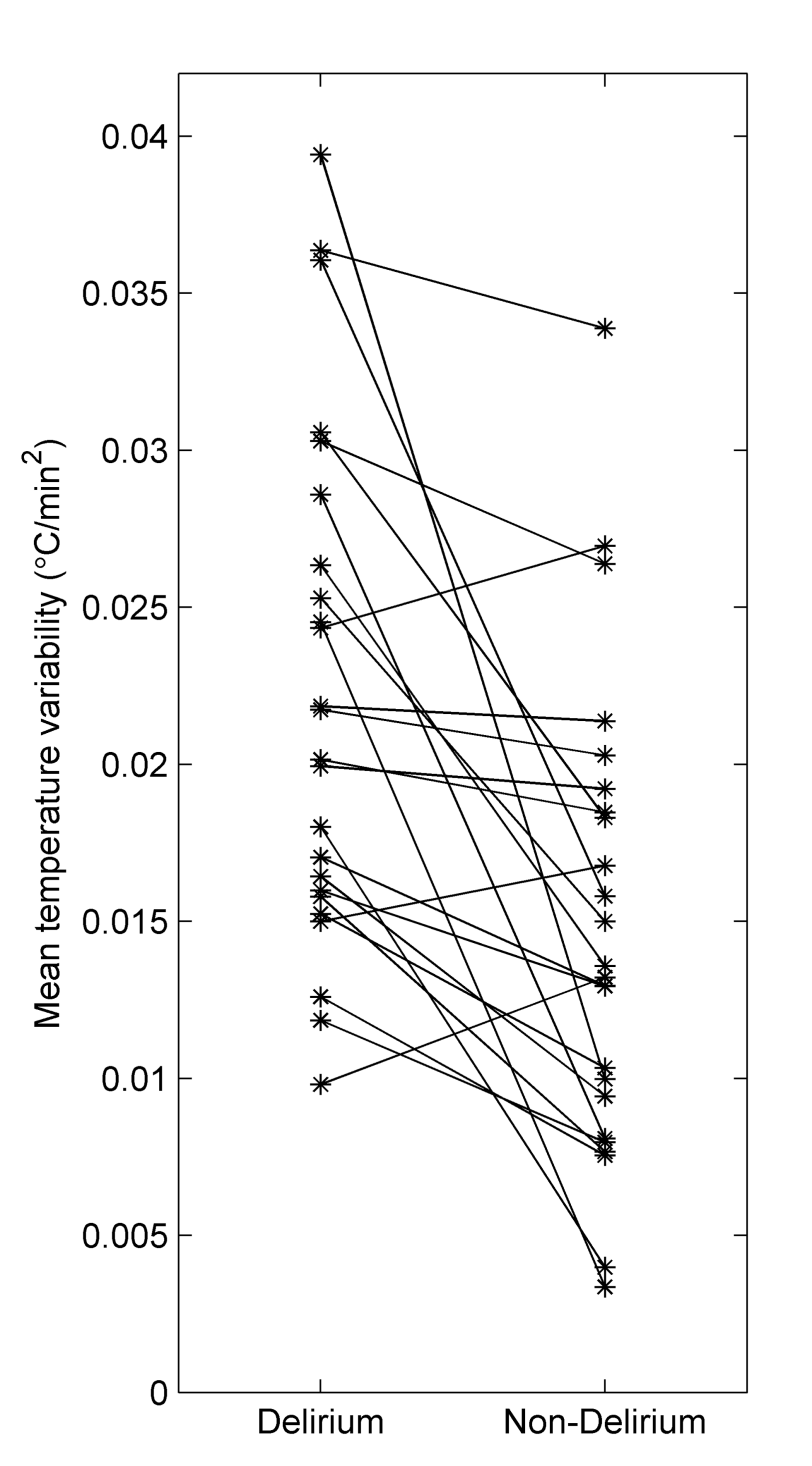
Temperature variability per patient. Per patient the mean temperature variability is depicted for delirium and non-delirium days. The majority of patients (21/24) shows higher mean temperature variability during delirium.

Overall, the median (interquartile range) of the number of samples per measurement day was 755 (506-1027). In Figure 2, the determination of temperature variability is explained for one patient. The differences per patient for temperature variability are shown in Figure 3. Of the 24 patients, 21 patients (88%) showed increased temperature variability during delirium when compared to non-delirium. The mean temperature variability on delirium days was 0.021 (Std 0.008) and non-delirium days 0.015 (Std 0.010). The best unadjusted and adjusted linear mixed models for temperature variability included only a random intercept and no random slopes. Both the unadjusted and adjusted linear mixed models showed that temperature variability is increased during delirium (β_unadjusted_=0.005, 95% CI=0.003 to 0.008, p<0.001 and β_adjusted_=0.005, 95% CI= 0.002 to 0.008, p<0.001). 

The mean absolute body temperature on delirium days was 36.9 °C (SD=0.50) and on non-delirium days 36.9 °C (Std=0.58). Of the 24 patients, 13 patients (54%) showed decreased temperature during delirium when compared to non-delirium. The best unadjusted and adjusted linear mixed models for absolute body temperature also included only a random intercept and no random variables. Both the unadjusted and adjusted linear mixed models showed that delirium is not associated with absolute body temperature (β_unadjusted_=-0.03, 95% CI=-0.17 to 0.10, p=0.61 and β_adjusted_=-0.03, 95% CI=-0.17 to 0.10, p=0.63). 

## Discussion

In summary, temperature variability was found to be increased in ICU patients during delirium-days when compared to days without delirium. When we adjusted for level of activity and disease severity, temperature variability remained increased during delirium. Absolute body temperature was not related to delirium.

Increased temperature variability during delirium can be a manifestation of the encephalopathy that underlies delirium. Using electroencephalography, it has been shown that during delirium the brain is suffering from an encephalopathy[23]. Body temperature is mainly controlled by the hypothalamus. Encephalopathic changes may affect the thermoregulation network of the hypothalamus, which impairs the capability to keep body temperature constant. In addition, the underlying etiology of delirium could also be a source for increased temperature variability. In Wernicke encephalopathy, delirium tremens and schizophrenia, thermal dysregulation has been described[14-16]. In ICU delirium, this has never been studied before.

This is the first study on temperature variability in ICU delirium. Previously, temperature variability has been investigated with approximate entropy, detrended fluctuation analysis and wavelet properties[24-26]. However, these variables are more complex to compute than the mean absolute second derivative of the temperature signal that was used in the present study. To investigate temperature curve complexity, a continuous temperature signal is needed, with minimal artifacts. In patients with hyperactive or mixed type delirium it can be difficult to establish continuous temperature measurements for several days, due to an increased amount of movements which may result in artifacts. Therefore, a less complex time based analysis method, which is less vulnerable for artifacts, was used in this study to investigate whether the temperature curve differs between delirium days and non-delirious days in the same patient. This time based analysis method can be easily implemented in a monitoring device.

Other strengths of this study are that delirium diagnoses were prospectively obtained per day by research-nurses and –physicians with a combination of the CAM-ICU and review of medical and nursing charts. Although in daily practice the sensitivity of the CAM-ICU for detecting delirium may be disappointing, in research setting the CAM-ICU proves to be a sensitive tool for detecting delirium with sensitivities of 64 to 100% and specificities of 88 to 100%[9,11,27]. Although, we excluded all patients with characteristics that may affect temperature variability, we still found increased temperature variability during delirium. 

 A limitation of this study was that temperature data was obtained retrospectively. To minimize artifacts one could argue to obtain the data prospectively in order to control the environment of the measurement. The measurement in the inguinal crease may have been biased due to influence of sedative medication on skin perfusion. We excluded deeply sedated and comatose patients and thereby the effects of deep sedation. In addition, we adjusted for the level of sedation (RASS) in our linear mixed model analysis, to minimize the effect of sedation on temperature variability. Correcting for this confounder did not alter our results. Moreover, delirious patients may be more likely to remove thermometers which could have affected our results. By using the RASS score as a variable in the linear mixed model, we aimed to adjust for the fact that awake patients will move more and are more likely to remove thermometers. Correction for this variable did not alter the results. Therefore, the effect seems minimal. A future prospective design could resolve this problem. 

As a first step to evaluate whether temperature variability could be used for delirium monitoring, we excluded patients with conditions affecting thermal regulation or therapies affecting body temperature as well as data of days on which the patient suffered from sepsis. One could argue to exclude several days before sepsis as well, as previous literature showed that delirium preceded the development of overt sepsis by at least 48 hours in 31% of all patients[28]. By using a linear mixed model and correcting for the Sequential Organ Failure Assessment score (also known as Sepsis-related Organ Failure Assessment) we corrected for organ faillure prior to possibly upcoming sepsis. Correcting for this confounder did not alter our results. In this study, we did not investigate whether individuals in the ICU who never developed delirium have the same temperature variability. Instead, to increase statistical power, we compared delirium days with days without delirium, where patients were their own controls. Of note, 15 of the 24 patients included in this study were treated with haloperidol. We cannot exclude that the administration of pharmacological substances has a certain influence on our findings. However, such an influence of haloperidol on temperature variability has never been described.

The natural circadian rhythm of body temperature also gives rise to a temperature variability which is on average 1.2*10^-5^ °C/min^2^ for a cosines with an amplitude of 1 degree Celsius[29]. Removal of time periods with signal containing artifacts could have influenced our findings. However, the circadian temperature variability was approximately a thousand times smaller than the temperature variability measured in this study. Therefore, this could only have a minimal effect on the difference in temperature variability between delirium- and non-delirium days.

Future prospective diagnostic studies should explore whether temperature variability can be used as a diagnostic parameter in an unselected population of ICU patients for delirium monitoring. These studies should also consider blood stream temperature measurement (via central line or artery), because this might lead to more reliable data. In this first explorative study, we could only investigate if there are any opportunities to use temperature variability for monitoring. When our findings are confirmed, temperature variability could be incorporated together with other physiological parameters into an objective monitoring device for delirium. Electroencephalography (EEG) shows generalized slowing of background activity during delirium[13]. Temperature variability together with EEG, with a limited number of electrodes and automatic processing, could provide the input for an objective tool to monitor delirium. 

Temperature variability is increased during delirium in ICU patients. Opportunities for delirium monitoring using temperature variability should be further explored. Particularly, in combination with EEG, it could provide the input for an objective tool to monitor delirium in ICU patients. 
